# Accuracy of commercially available c-reactive protein rapid tests in the context of undifferentiated fevers in rural Laos

**DOI:** 10.1186/s12879-016-1360-2

**Published:** 2016-02-04

**Authors:** Koukeo Phommasone, Thomas Althaus, Phonesavanh Souvanthong, Khansoudaphone Phakhounthong, Laxoy Soyvienvong, Phatthaphone Malapheth, Mayfong Mayxay, Rebecca L. Pavlicek, Daniel H. Paris, David Dance, Paul Newton, Yoel Lubell

**Affiliations:** 1 Lao-Oxford-Mahosot Hospital-Wellcome Trust Research Unit (LOMWRU), Mahosot Hospital, Vientiane, Laos; 2 Faculty of Postgraduate Studies, University of Health Sciences, Vientiane, Laos; 3 Mahidol-Oxford Tropical Medicine Research Unit (MORU), Faculty of Tropical Medicine, Mahidol University, Bangkok, Thailand; 4 Nuffield Department of Medicine, Centre for Tropical Medicine and Global Health, University of Oxford, Oxford, UK; 5 Naval Medical Research Center-Asia, Embassy of the United States of America, Singapore, Singapore

**Keywords:** C Reactive Protein, Diagnostic accuracy, Southeast Asia, Laos

## Abstract

**Background:**

C-Reactive Protein (CRP) has been shown to be an accurate biomarker for discriminating bacterial from viral infections in febrile patients in Southeast Asia. Here we investigate the accuracy of existing rapid qualitative and semi-quantitative tests as compared with a quantitative reference test to assess their potential for use in remote tropical settings.

**Methods:**

Blood samples were obtained from consecutive patients recruited to a prospective fever study at three sites in rural Laos. At each site, one of three rapid qualitative or semi-quantitative tests was performed, as well as a corresponding quantitative NycoCard Reader II as a reference test. We estimate the sensitivity and specificity of the three tests against a threshold of 10 mg/L and kappa values for the agreement of the two semi-quantitative tests with the results of the reference test.

**Results:**

All three tests showed high sensitivity, specificity and kappa values as compared with the NycoCard Reader II. With a threshold of 10 mg/L the sensitivity of the tests ranged from 87–98 % and the specificity from 91–98 %. The weighted kappa values for the semi-quantitative tests were 0.7 and 0.8.

**Conclusion:**

The use of CRP rapid tests could offer an inexpensive and effective approach to improve the targeting of antibiotics in remote settings where health facilities are basic and laboratories are absent. This study demonstrates that accurate CRP rapid tests are commercially available; evaluations of their clinical impact and cost-effectiveness at point of care is warranted.

## Background

In most febrile patients presenting with non-specific symptoms an accurate clinical diagnosis is not possible. This is particularly challenging in the presence of diverse aetiologies and inadequate diagnostic facilities, characteristic of low income, malaria-endemic settings. While malaria can be diagnosed with the aid of rapid diagnostic tests (RDTs), the majority of fevers across much of the malaria-endemic world have other causes [1–5]. These patients commonly receive antibiotics when they are not indicated, often for mild, self-limiting viral infections. Conversely, antibiotics are often withheld from patients they could benefit. This irrational and poorly targeted use of antibiotics is a known contributor to the ever increasing threat of antimicrobial resistance that plagues global health [6].

Improving the management of non-malarial fevers is also crucial for malaria elimination campaigns that necessitate diagnosis and treatment of all remaining malaria cases. In many areas in Asia community health worker networks are being extended to support this aim. The utilization of their services, however, is likely to suffer if they are unable to influence the care of the majority of patients with non-malarial causes of fever.

C-reactive protein (CRP) is a non-specific marker of inflammation. Use of CRP point-of-care tests to guide the use of antibiotics in fevers and respiratory infections in primary care is standard practice in a number of high-income countries [7–10]. A recent analysis of over 1400 samples from fever studies in the Mekong region confirmed the ability of CRP to distinguish between viral and bacterial infections in these settings [11]. The quantitative readers used in high-income facilities, however, might not be practical or affordable in rural tropical settings. We evaluated three commercially available CRP immunochromatographic rapid tests of a format that could be used by community health workers in remote settings to improve the management of non-malarial fevers. This evaluation aims to provide a general assessment of the reliability of the existing technology underlying these tests to correctly classify patients as having CRP values above or below the rapid test threshold(s). Whether or not CRP is an effective guide for antibiotic treatment and the identification of appropriate threshold(s) to inform patient management is the subject of further clinical outcome studies.

## Methods

Samples for this investigation were obtained from a fever study in rural Laos aiming to provide a prospective description of the clinical features and aetiology of fever among consecutive outpatients attending three provincial hospitals in the provinces of Salavan, Luang Namtha, and Xieng Khouang over one year. Adults giving informed consent or children (<18 years) whose parent or guardian gave informed consent who presented with a history of fever for ≤8 days and/or admission body temperature ≥38°c were recruited. CRP readings were not used to inform patient recruitment or management as the role of CRP in guiding antibiotics in these settings is still under investigation.

Two individuals carried out independent searches for commercially available CRP rapid tests with analytical ranges and thresholds relevant to the detection of bacterial infections (>10 mg/L). Three of five available tests were evaluated in this study, with two further tests under current evaluation in another study. In each site one of the three CRP RDTs and the reference test was performed at point-of-care immediately after patient presentation. Two of the sites used serum for both the RDT and the reference test (DTS233, Creative Diagnostics, USA; WD-23, Assure Tech, China). In a third site whole blood was used for the RDT (bioNexia CRPplus, bioMérieux S.A., France) and EDTA plasma was used for the reference test (see Table 1 for test characteristics). All tests were performed and interpreted by a laboratory technician according to the manufacturers’ instructions. In all sites a NycoCard Reader II (Alere/Axis-Shield, Oslo, Norway) for quantitative CRP readouts was used as a reference test. The NycoCard Reader II has an analytical range of 5-120 mg/L using serum/plasma or 8-120 mg/L in whole blood and its accuracy has previously been confirmed to be high [12], including in the context of resource-poor settings [13]. Due to limited study personnel at the sites, the RDTs and the reference tests were carried out in an unblinded manner by the same individual, with the RDT being interpreted prior to the reference test to minimise the risk of bias.

**Table 1:** Rapid test characteristics

	Manufacturer	Recommended reading time	Format and thresholds	Visual interpretation
DTS233	Creative Diagnostics, USA	10 minutes, no more than 15 minutes.	Qualitative, single threshold of 10mg/L	
WD-23	Assure Tech, China	5 minutes; no more than 7 minutes.	Semi-quantitative; <10mg/L; 10-30mg/L; 30-80mg/L; >80mg/L	
				A positive result and the classification determined by the appearance and relative strength of the test band as compared with the control and reference bands.
bioNexia CRPplus	bioMerieux, France	Exactly 5 minutes	Semi-quantitative; <10mg/L; 10-40mg/L; 40-80mg/L; >80mg/L	

### Statistical analysis

The accuracy of the RDTs was calculated with respect to the thresholds stated by each manufacturer. All three tests shared a threshold of 10 mg/L to define positivity. The two semi-quantitative tests had additional higher thresholds, of either 30 or 40 mg/L, and of 80 mg/L. We calculated the sensitivity and specificity of each of the three tests with a binary interpretation of the result using the shared threshold of 10 mg/L. Sensitivity was calculated as the number of individuals with a positive RDT out of all patients with a reference test result >10 mg/L and specificity as the number of individuals with negative RDT out of all those with a reference test result of <10 mg/L. Confidence intervals for sensitivity and specificity were calculated using the Wilson score method. For the two semi-quantitative tests with multiple thresholds we categorised patients according to their reference test result using the RDT manufacturers’ specified thresholds, and used a weighted Kappa statistic for the agreement in RDT classification as compared with the NycoCard Reader II results [14]. The analysis was carried out using Stata 14 (StataCorp. Texas, USA).

Ethical approval was obtained for the fever study from the Lao National Ethics Committee for Health Research and from the Oxford Tropical Ethics Committee (OXTREC, reference 27–14). Both bodies also approved an amendment to the original protocol for this sub-study. The Informed Consent form allowed for samples to be used for microbiological and diagnostic investigations.

## Results

From the introduction of the RDTs in March 2015 up until the end of July 2015, a total of 837 patients were recruited into the fever study. Demographic and clinical characteristics of these patients are described in Table 2. In all sites fever was only present in one third of patients at enrolment (35, 25.6, and 39.2 % in Salavan, Luang Namtha and Xieng Khouang respectively). Respiratory syndrome was the most frequent presentation, occurring in 81.7 % of cases (82.1 % in Salavan, 85.2 % in Luang Namtha, and 75.6 % in Xieng Khouang).

**Table 2:** Demographic and clinical characteristics of patients at the baseline, according to study site

Patients at baseline n=837	Sites in rural Laos		
	Salavan (n=223)	Luang Namtha (n=379)	Xieng Khouang (n=S235)
*Demographic characteristics*			
Male, n(%)	132 (59.2)	196 (51.7)	111 (47.2)
Age, median (IQR)	9 (1-27)	6 (1-23)	7 (1-25)
BMI (Kg/m^2^), mean (SD)	17.8 (3.88)	17.3 (4.23)	18.1 (4.16)
Pre-admission antibiotics consumption	34 (15.3)	118 (31.1)	56 (23.8)
Current antibiotics prescription, n (%)	98 (44.0)	273 (72.0)	121 (51.5)
-Penicillin M & V, n [%)	6 (6.1)	6 (2.2)	27 (22.3)
-Penicillin A, n (%)	31 (31.6)	225 (82.4)	19 (15.7)
-Cephalosporins, n (%)	20 (20.4)	13 (4.8)	25 (20.7)
-Quinolones, n (%)	24 (24.5)	14 (5.1)	8 (6.6)
-Macrolides, n (%)	7 (7.1)	11 (4.0)	10 (8.3)
-Others® [%)	10 (10.2)	4 (1.5)	32 (26.5)
Time (in days) between Onset of symptoms & Admission	3 (2-4)	3 (2-5)	2 (1-4)
*Clinical characteristics*			
Fever, n (%)*	78 (35.0)	97 (25.6)	92 (39.2)
Neurological presentation, n (%)**	105 (47.1)	220 (58.2)	180 (76.6)
Respiratory syndrome, n(%)***	183 (82.1)	322 (85.2)	177 (75.6)
Enteric syndrom, n (%)****	63 (28.3)	174 (46.0)	144 (61.3)
Undifferentiated fever, n (%)*****	6 (8.2)	4 (2.7)	4 (4.9)

* Fever defined by tympanic temperature >37.7°C

** Neurological syndrome includes the presence of at least one of these symptoms: stiffneck; cephalalgia; confusion

*** Respiratory syndrome includes the presence of at least one of these symptoms: lung crepitation; cough; dyspnoea

**** Enteric syndrom includes the presence of at least one of these symptoms: abdominal pain; vomiting; nausea; jaundice; diarrhoea

****** Undifferentiated fever includes the absence of any focal symptoms, coupled with the presence of at least one of these symptoms: fever; chills; rigors; myalgia

The overall prescription rate of antibiotics was 58.8 % in all sites (44 % in Salavan, 72 % in Luang Namtha, and 51.5 % in Xieng Khouang). Overall, penicillin A was the most prescribed antimicrobial in 55.9 % of cases, followed by cephalosporins (11.8 %). The Xieng Khouang site also had the highest rate of pre-attendance antibiotics consumption (31.1 %), compared with Salavan and Luang Namtha (15.3 and 23.8 % respectively).

**Fig. 1:**
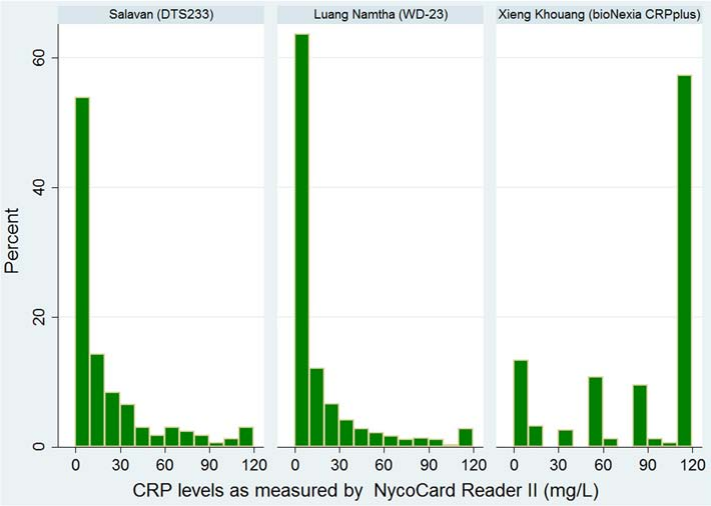
Distribution of CRP levels in the three sites

**Fig. 2:**
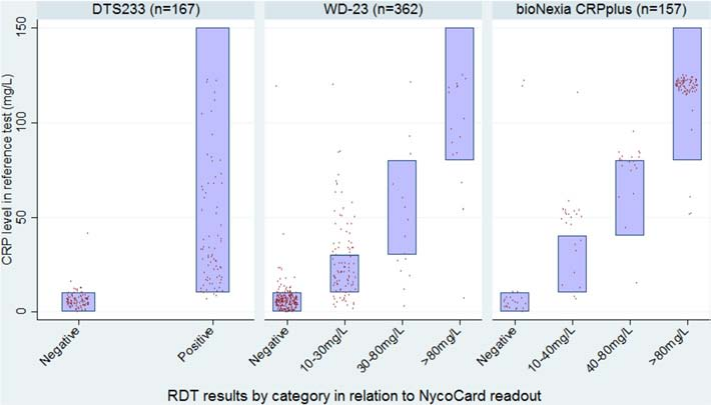
Results rapid tests in relation to the quantitative reference test

Of these 837 patients, 82 % had fresh-blood samples taken for CRP assays for immediate testing, at point-of-care. The distributions of CRP levels in the three sites according to the reference test are shown in Fig. 1. In two of the sites (Salavan and Luang Namtha) the majority of patients had CRP levels <10 mg/L, while in Xieng Khouang 86 % of patients had CRP levels above this threshold. A total of 167, 363 and 157 tests were carried out for the DTS233, WD-23 and bioNexia CRPplus tests, respectively. These data are plotted in Fig 2, along with the manufacturer specified ranges with which each test result is supposed to correspond.

Using the shared threshold of 10 mg/L and a binary interpretation of the RDT results (either negative or positive regardless of positivity category in the semi-quantitative tests), all three RDTs had high sensitivity and specificity, as shown in Table 3. The two semi-quantitative tests also showed high agreement with the reference test result, with weighted kappa values of 0.7 and 0.8 for the WD-23 and the bioNexia CRPplus tests, respectively.

**Table 3:** Accuracy of the three rapid tests as compared with the reference test

	Sensitivity at a threshold of 10mg/L (CI)	Specificity at a threshold of 10mg/L (CI)	Unweighted kappa	Weighted kappa
DTS233	95% (87%-99%)	98% (92%-100%)	0.93 (51%)	NA
CRP-W23	87% (79%-92%)	91% (87%-95%)	0.62 (80%)	0.7 (93%)
bioNexia CRPplus	98% (95%-100%)	91% (71%-99%)	0.68 (81%)	0.8 (93%)

## Discussion

Use of CRP rapid tests in remote settings could help ensure that patients in need of antibacterial treatment are identified as such, while reducing unnecessary use of antimicrobials in viral infections. All three tests included in this analysis exhibited high accuracy in line with their manufacturers’ specified thresholds. Other tests are available on the market; the selection of the three tests was arbitrary, based on their accessibility to our research units in Thailand and Laos.

All three tests had high accuracy when interpreted in a binary manner. The semi-quantitative tests also performed well in classifying patients into the specified range. In doing so they provide richer information that could feed into more nuanced algorithms to guide the use of antibiotics and decisions on referral. On the other hand, the interpretation of semi-quantitative tests can be more challenging and in the absence of a dedicated reader a binary test might be preferred.

Whether a single threshold is sufficient and the optimal value(s) to indicate the need for antibiotics and/or referral is not well established. The threshold of 10 mg/L is lower than most recommendations in high income settings [7, 15], although for remote settings with limited ability to follow patients up and obtain emergency care it might be appropriate to accept a higher rate of false positives. Further studies of CRP-guided treatment algorithms are required to identify optimal threshold(s).

There are limitations to this study. First, the RDTs used fresh serum and EDTA whole blood whereas if implemented in routine care CRP RDTs are likely to be performed on fresh whole blood. Second, in one site the reference test used a different matrix than the RDT. Third, due to restricted study personnel, the RDTs and the reference test were performed by the same individual. Further work is being carried out on a broader range of tests to address these limitations. Fourth, while patients were recruited prospectively into the study, in one site many children did not have a blood sample taken. This might partly explain the elevated CRP levels in this site as compared with the other two sites; consultations of children in primary settings often occur at an earlier stage than adults because of parental behaviour [16]. The limited number of individuals with low levels of CRP in this site resulted in a wide confidence interval of 71–99 % for the specificity of the RDT. Lastly, the NycoCard Reader II was used as a reference test although ELISA testing in reference laboratories might have provided more accurate measurements. The NycoCard Reader has, however, been used in similar remote settings, with excellent correlation with laboratory-based CRP [13].

Several questions are highlighted for further research. The use of rapid CRP tests needs to be prospectively evaluated in routine settings, including estimating their accuracy using fresh whole blood; inter-observer agreement; and the importance of timing. In a previous evaluation of CRP tests it was shown that even small delays in reading the test results can be detrimental to their accuracy [17]. Most important, however, are investigations into the clinical implications of CRP-guided treatment algorithms, to gauge whether they can safely reduce antibiotic prescriptions without impacting adversely on outcomes. The thresholds used in currently available qualitative or semi-quantitative tests might not be optimal for these purposes; further clinical and economic evaluations are required to identify optimal thresholds. Whether health workers and patients adhere to CRP test results will also need to be determined. Notwithstanding these questions, this analysis provides further support to the possible implementation of CRP rapid tests at point-of-care, in order to inform the management of febrile patients with a negative malaria test.

## Conclusions

Over the last few years, widespread use of malaria rapid test transformed the management of fevers in tropical settings. Improved targeting of antimalarials, however, has been accompanied by increasing misuse of antibiotics, often prescribed to patients they will not benefit, while treatable infections go undetected. There is increasing evidence from aetiological and clinical studies indicating that CRP testing could contribute to better identification of patients that require treatment, while reducing overall drug pressure and mitigate the spread of antimicrobial resistance.

This study evaluated simple lateral flow CRP tests that could potentially be used in the most remote settings to guide referral and treatment of non-malarial fevers. The three tests performed well as compared with a quantitative validated reader in detecting elevated levels of CRP. While further research is required into the performance of such tests in field conditions and to determine the optimal cut-off threshold(s) for CRP guided treatment, the timeframe to introduction of such tests could be relatively short. New pathogen specific multiplex testing and/or other biomarkers could again revolutionize the management of fever in the long run, but until these become available current practice can and should evolve using available tests that are already routinely used in other settings.

## Abbreviations

CRP: C-Protein reactive
RDT: Rapid Diagnostic Test
